# Structural and Biochemical Investigation of Bacteriophage N4-Encoded RNA Polymerases

**DOI:** 10.3390/biom5020647

**Published:** 2015-04-27

**Authors:** Bryan R. Lenneman, Lucia B. Rothman-Denes

**Affiliations:** 1 Committee on Genetics, Genomics, and Systems Biology, The University of Chicago, 920 East 58th Street, Chicago, IL 60637, USA; 2 Department of Molecular Genetics and Cell Biology, The University of Chicago, 920 East 58th Street, Chicago, IL 60637, USA

**Keywords:** bacteriophage N4, single-subunit polymerases, N4 virion RNA polymerase, N4 RNAPII

## Abstract

Bacteriophage N4 regulates the temporal expression of its genome through the activity of three distinct RNA polymerases (RNAP). Expression of the early genes is carried out by a phage-encoded, virion-encapsidated RNAP (vRNAP) that is injected into the host at the onset of infection and transcribes the early genes. These encode the components of new transcriptional machinery (N4 RNAPII and cofactors) responsible for the synthesis of middle RNAs. Both N4 RNAPs belong to the T7-like “single-subunit” family of polymerases. Herein, we describe their mechanisms of promoter recognition, regulation, and roles in the phage life cycle.

## 1. Introduction

Regulation at the transcriptional level is the primary means used by bacteriophage to progress through distinct developmental stages during infection. Gene products expressed immediately after infection are primarily involved in the takeover of essential host processes. Predominantly, phage utilize the host RNAP to recognize the phage early promoters; subsequently, a product of phage early transcription either modifies the properties of the host RNAP to overcome transcription termination signals (*i.e*., λN, λQ, HKO22 Put RNA), redirects the host RNAP to middle promoters through sigma factor remodeling (*i.e*., T4 AsiA-MotA) or replaces the host vegetative sigma factor by phage-encoded sigma factors that direct the transcription of middle and late genes (*i.e*., SPO1 gp28, gp34) [1,2,3]. Alternatively, host RNAP transcription of the phage early genes results in the synthesis of a phage early gene product (*i.e*., ø29 gp4) that activates the host RNAP to utilize the phage weak late promoter while inhibiting the transcription of the phage early genes, eliciting the transition from early to late transcription [4]. In all above-mentioned cases, the host RNAP core enzyme is essential throughout the phage growth cycle. Host RNAP transcription of the early genes of other phage leads to the synthesis of host RNAP inhibitors (*i.e*., T7 gp2, *Xanthomonas oryzae* phage Xp10 P7) and of a phage-encoded RNAP whereby the phage becomes transcriptionally independent of the host (*i.e*., T7 RNAP, Xp10 RNAP) [5,6,7,8]. Middle transcription then commences, focusing largely on the synthesis of proteins involved in phage genome replication. Genome replication is followed by late gene transcription, which includes the production of both morphogenetic proteins, involved in virion assembly and DNA packaging, and of proteins required for host cell lysis. In contrast, the recently described *Pseudomonas aeruginosa* giant bacteriophage øKZ provides a unique example of a transcriptional strategy that is completely independent of the host. Two sets of phage-encoded polypeptides that have homology to the β and β' subunits of bacterial RNAPs have been identified, with one set present in virions. Three classes of putative promoter sequences have been identified upstream of genes transcribed at early, middle, and late times during infection. The role of these polypeptides and sequences in transcription of the phage genome has not yet been confirmed [9].

**Figure 1:**
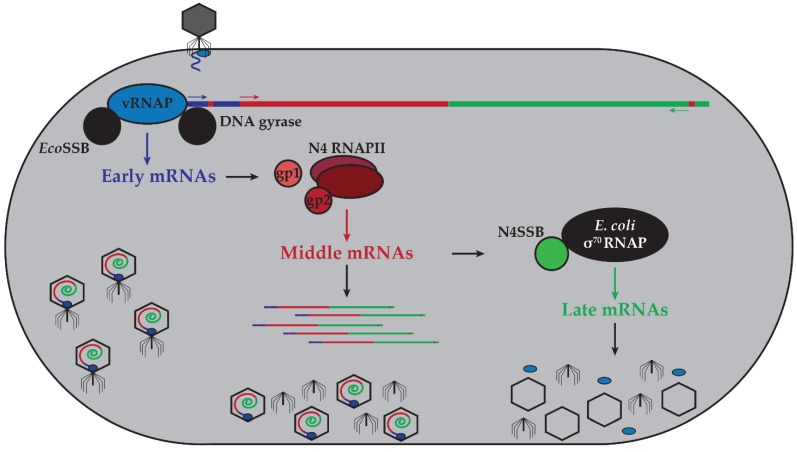
Schematic of transcriptional program controlling the N4 life cycle. Upon interaction with its receptor, NfrA, N4 injects vRNAP and genomic DNA into the host. Host DNA gyrase introduces negative supercoils into the phage genome, driving early promoter hairpin extrusion. *Eco*SSB stabilizes the hairpin structures, allowing vRNAP to initiate transcription from early promoters to transcribe early genes required for middle transcription and transport the genome into the host cytoplasm. The early gene products gp1 and gp2 act as cofactors for the heterodimeric polymerase N4 RNAPII (gp15 and gp16) to carry out middle transcription. These transcripts encode functions required for N4 DNA replication. One such protein, N4SSB, interacts with host σ^70^-RNAP, redirecting it to late promoters. The late genes encode morphogenetic proteins involved in virion assembly, DNA and vRNAP packaging, and host lysis. Host proteins depicted in black, phage-encoded early transcriptional machinery in blue, phage-encoded middle transcriptional machinery in shades of red, and phage-encoded late transcriptional machinery in green. Arrows indicate polarity of transcription.

The *Escherichia coli* K-12 strain-specific bacteriophage N4, isolated from the sewers of Genoa, has evolved a unique and “reversed” transcriptional strategy (Figure 1). N4 establishes transcriptional independence of the host immediately upon infection through the injection of a virion-encapsidated RNAP that transcribes the phage early genes. This independence is maintained through middle transcription catalyzed by a second phage-encoded RNAP and its cofactors, which transcribe genes encoding phage replicative functions. In contrast to the previous transcriptional strategies described above, late N4 transcription is carried out by the host σ^70^-RNAP directed to the late promoters by N4 single-stranded DNA-binding protein (N4SSB), essential for N4 DNA replication.

## 2. N4 Transcriptional Architecture

### 2.1. N4 vRNAP Synthesizes N4 Early RNAs

Unlike most phage, where the host RNAP holoenzyme is responsible for the synthesis of their early mRNAs, a burst of RNA synthesis is observed immediately after N4 infection under conditions where the host RNAP is inhibited [10]. This transcription occurs even under conditions where post-infection protein synthesis is inhibited, suggesting the existence of a novel activity and leading us to postulate the presence of a RNAP in N4 virions [10,11]. This hypothesis was confirmed by detecting a RNAP activity in extracts from purified N4 virions disrupted through a denaturing protocol [12,13]. A single polypeptide of molecular weight 320 kDa was purified to homogeneity [13]. *In vitro* analysis showed that the protein requires the four ribonucleotide triphosphates, Mg^2+^, and denatured N4 DNA as a template for RNA synthesis [12,13,14]. vRNAP was completely inactive on native N4 DNA, but transcribed denatured N4 DNA efficiently and, most notably, with *in vivo* specificity. In contrast with other RNAPs, N4 vRNAP cannot use other denatured DNA templates (T4, T7, salmon sperm) [12,14,15,16].

Given the inactivity of vRNAP on native N4 DNA and its preference for single-stranded DNA, we proposed that the structure of phage DNA might become modified upon its injection into the host, rendering it competent for transcription by vRNAP [12,14]. Host DNA gyrase, which introduces negative superhelical turns into circular DNA, was tested for its involvement in N4 early RNA synthesis [17]. Treatment of cells prior to N4 infection with coumermycin, an inhibitor of *E. coli* DNA gyrase, significantly reduced N4 early transcription *in vivo*, suggesting that host DNA gyrase, and therefore negative supercoiling, is required for vRNAP cognate promoter recognition [14,18]. Considering that the N4 double-stranded DNA genome is linear, these results are surprising but can be explained if the genome is topologically constrained during injection. In support of this mechanism, the leftmost portion of the genome containing all vRNAP promoters is injected first [19].

To elucidate the template requirements for specific vRNAP transcription, sites of transcription initiation were mapped both *in vivo* and *in vitro* to the nucleotide level, revealing three sites of vRNAP initiation (P1, P2, and P3) within the leftmost 10% of the genome [19,20,21]. Sequences spanning −17 to +1 (relative to the transcription start site at +1) are conserved across all three promoters and include a GC-rich heptamer centered at −11 flanked by inverted repeats [21]. The conservation of inverted repeats, coupled with the requirement of host DNA gyrase *in vivo*, suggests a model where vRNAP transcription depends on the supercoil-induced extrusion of hairpin sequences mediated by DNA gyrase.

Subsequent studies confirmed key features of this model. The conserved hairpin stem contains both conserved and variable nucleotides. The conserved bases in all three promoters were shown to be required for both vRNAP binding and transcription initiation, while the identity of the non-conserved bases was shown to be extraneous as long as they maintain the promoter hairpin, suggesting that hairpin formation and direct contacts with vRNAP are required for promoter recognition [22]. Hairpin structure was detected by the cleavage patterns of single-stranded DNA templates upon treatment with enzymatic and chemical probes [22]. Although these data clearly prove the presence and function of hairpins in vRNAP promoters using *in vitro* systems, theoretical predictions of hairpin extrusion suggested that short hairpins can only form at superhelical densities significantly greater than those observed under physiological conditions [23,24]. Circles of phage DNA with differing superhelical densities and containing two early promoters were generated and probed for hairpin extrusion by four different chemical and enzymatic methods. DNA cruciform structures were detected at sub-physiological superhelical densities (−0.035) in the presence of Mg^2+^ [25]. The hairpins were shown to form *in vivo* by introducing the promoter P1 hairpin sequence between the −35 and −10 regions of the *E. coli rrnB* gene promoter. In this strain, promoter activity was abolished, while inhibiting DNA gyrase with the drug novobiocin restored activity [25]. These results clearly demonstrate that the N4 promoter hairpin is not only capable of extrusion under physiological conditions, but that extrusion occurs *in vivo* in a DNA gyrase activity-dependent manner.

**Figure 2:**
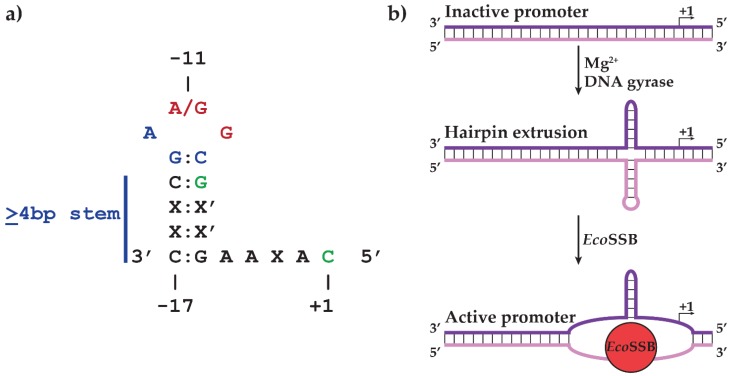
(**a**) Summary of sequence requirements for early promoter hairpin-extrusion (blue), vRNAP binding (green), and both processes (red) as determined by hairpin extrusion probing and runoff transcription experiments. Template strand sequences are shown relative to the transcription start site (+1) in 3' to 5' orientation. (**b**) Model of early promoter activation. N4 genomic DNA is injected into the host cytoplasm in an inactive linear double-stranded conformation. Introduction of negative supercoils by DNA gyrase induces the extrusion of the promoter hairpin in a Mg^2+^- and sequence-dependent manner. Subsequent binding of *Eco*SSB leads to melting of the non-template strand hairpin and stabilization of the template strand hairpin to provide the active promoter conformation required for vRNAP binding. Template and non-template strands of promoter DNA are represented as purple and pink, respectively.

The unexpected ability of N4 promoter hairpins to extrude at physiological DNA superhelical densities led to a series of experiments to determine the DNA sequence requirements for this process. The results of runoff transcription and hairpin-extrusion probing on promoter mutant templates are summarized in Figure 2a. The observation that the non-template strand hairpin loop was sensitive to single–stranded probes while the template strand loop was resistant was surprising and indicated structural differences determined by the loop sequences between the two DNA strands [25,26]. Analysis of the sequence requirements revealed that the 3'G:C5' loop closing base pair and the 3'A and 5'G bases of the loop are required for hairpin extrusion and hairpin stability [22,25,26]. Results of runoff transcription assays on mutant promoters and of crosslinking experiments with templates containing the photocrosslinking nucleotide analog 5-iododeoxyuracil (5-IdU) at specific positions showed that vRNAP specifically recognizes −11A/G, −10G, −8G, and +1C [26,27,28]. Contact with the −8 base was shown to occur through the major groove of the hairpin stem, while the site of crosslinking to the −11 purine residue was mapped to W129 in vRNAP [28].

The initial model of promoter recognition by vRNAP involved the introduction of negative supercoils into N4 DNA concurrent with genome injection. However, supercoiled promoter-containing plasmids were not active templates for vRNAP transcription *in vitro*, suggesting the requirement of another factor for promoter activation. Genetic analysis identified the missing component as *E. coli* single-stranded DNA-binding protein (*Eco*SSB). No *in vivo* transcription of early promoters contained within plasmids in *ssb-1* (ts) mutant hosts was detected at the restrictive temperature [29]. *In vitro*, *Eco*SSB activates vRNAP transcription initiation 40-fold from supercoiled templates with *in vivo* specificity [29]. This process is due to invasion and melting of the non-template strand hairpin while the template strand hairpin is not perturbed, an unexpected behavior since single-stranded DNA binding proteins’ role is to erase DNA secondary structures [29,30,31]. In this context, *Eco*SSB is acting as an architectural transcription factor by providing an active promoter conformation for vRNAP binding (Figure 2b) [27].

Along with its role in promoter activation, *Eco*SSB plays a role in transcript displacement. vRNAP transcription on single-stranded templates leads to RNA:DNA hybrid formation. At limiting template concentrations, addition of *Eco*SSB activates transcription [29]. S1 nuclease protection assays in reactions containing *Eco*SSB showed that the protein binds to the transcript as it exits vRNAP and facilitates template recycling *in vitro* [29,32]. Interaction of *Eco*SSB with the emerging transcript was confirmed by the results of crosslinking experiments with transcripts containing 5-IdU substitutions at specific positions [32]. Comparison of the T7 RNAP and N4 vRNAP sequences indicated that part of the T7 RNAP N-terminal domain responsible for RNA separation and exit is missing from vRNAP; therefore, we have proposed that *Eco*SSB fulfills this role in vRNAP transcription [32,33]. Our inability to detect *Eco*SSB-N4 vRNAP interactions with purified proteins suggests that these might occur as the polymerase transitions into the elongation complex.

The large size (3500 aa, 320 kDa) of vRNAP suggests that it may have multiple functions in phage development, along with several domains responsible for each activity. In order to define the minimally active transcriptional domain of vRNAP, the protein was subjected to limited trypsin digestion. A 1106 aa (998–2103), 122 kDa domain was identified that possesses the same transcription initiation, elongation, and termination properties as the full-length polypeptide [34]. A BLAST search with the minimally active transcriptional domain (mini-vRNAP) of vRNAP showed that this protein belongs to the family of T7-like “single-subunit” RNAPs, which encompasses the bacteriophage-encoded, nuclear-encoded mitochondrial and chloroplast, and linear plasmid-encoded enzymes [34,35]. Mini-vRNAP represents a highly-diverged member of this family, with little sequence homology outside of the conserved sequence blocks that have been implicated in catalysis [34]. Indeed, motifs A, B, and C, along with the DX_2_GR (TX_2_GR in mini-vRNAP) motif are all present within mini-vRNAP and mutational analyses show that their catalytic functions are conserved between T7 RNAP and mini-vRNAP [34].

**Figure 3:**
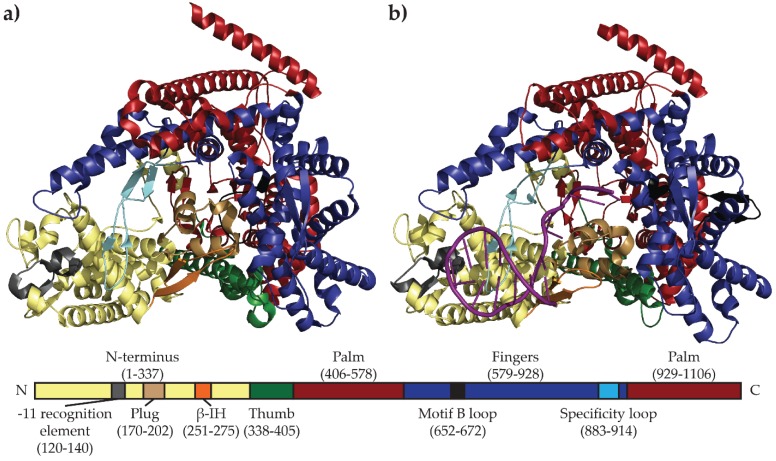
Comparison of the mini-vRNAP apo (Accession number 2PO4) (**a**) and BC (Accession number 3C3L) (**b**) structures highlighting rearrangements near the enzyme active site. Both structures show “cupped right hand” architecture (thumb, palm, fingers) characteristic of this family of RNAPs. Upon promoter binding, large scale structural rearrangements of the plug and β-IH motif occur, allowing the rearrangement of the motif B loop into the O helix to allow single-stranded DNA access to the active site. Bottom bar represents the mini-vRNAP primary sequence with amino acid numbering indicated in parentheses. Domains and structural motifs are labeled and colored as in the crystal structures, with template strand DNA represented as a purple ribbon.

Despite the low level of sequence similarity between mini-vRNAP and T7-like polymerases, they share a common architecture. The “cupped right hand” architecture shared by all related DNA and RNA polymerases is evident in the crystal structure of the apo form of mini-vRNAP, solved at 2.0 Å resolution [36]. A comparison with the crystal structure of T7 RNAP shows that the active sites of T7 RNAP and mini-vRNAP superimpose, reinforcing results of mutational analyses [34,36,37]. This comparison also identified three structural motifs in mini-vRNAP shown to be required for promoter recognition in T7 RNAP: the AT-rich recognition loop, β-intercalating hairpin (β-IH), and specificity loop [38,39]. Surprisingly, the crystal structure of apo mini-vRNAP reveals two structural motifs, the “plug” and the “motif B loop,” that block the pathway of the DNA to the active site, suggesting that the structure represents an inactive conformation (Figure 3a) [36].

This model was confirmed by comparing (Figure 3) the apo structure with that of the binary complex (BC) of mini-vRNAP and its promoter P2 solved at 2.4 Å resolution (Figure 3b). The structures show a 25.1° rotation of the plug and β-IH motif, allowing for a 32.6 Å movement of the motif B loop and its rearrangement into the O helix upon promoter binding, which then grants single-stranded DNA access to the active site [36,40]. Crosslinking studies tethering the plug inside the active site resulted in transcriptionally inactive enzymes, confirming the necessity of these rearrangements [40]. The apo and BC mini-vRNAP structures present a model where vRNAP is present in an inactive conformation until it recognizes its promoter hairpin, explaining why vRNAP does not transcribe single-stranded DNA templates devoid of promoter hairpins. This DNA conformation is an allosteric effector that acts as a key to unlock the pathway of the template to the active site and render vRNAP competent for transcription initiation.

**Figure 4:**
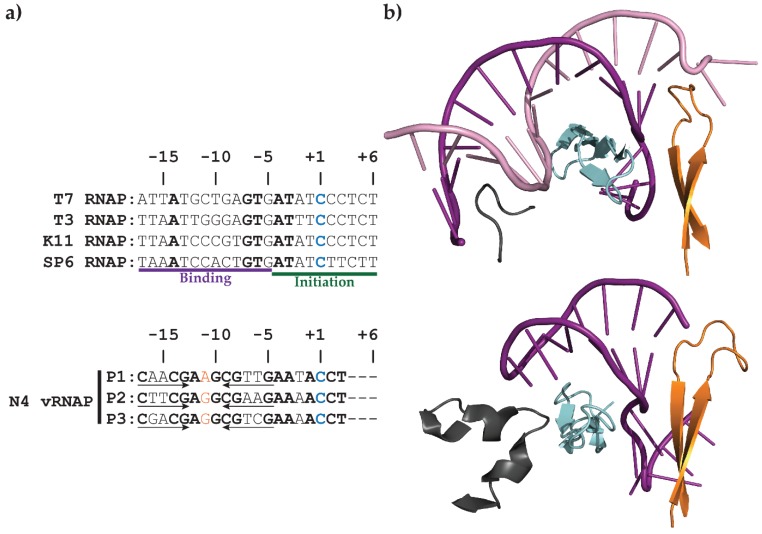
(**a**) Consensus promoter sequences of T7 and other related phage RNAPs compared with N4 vRNAP promoters. Template strand sequences spanning −17 to +6 are shown relative to the transcription start site (+1) in 3' to 5' orientation. Bold: conserved bases in all promoters both within and across species. Blue: +1 nucleotide. Arrows: inverted repeats. Orange: central base of hairpin loop. Purple underline: −17 to −5 binding region. Green underline: −4 to +6 initiation region. (**b**) Comparison of the three structural motifs responsible for promoter recognition between T7 RNAP (Accession number 1CEZ) (top) and mini-vRNAP (bottom). The AT-rich recognition site (T7 RNAP) and −11 recognition element (mini-vRNAP) (grey) are responsible for recognition of upstream sequences. The specificity loops (cyan) recognize the promoter by sequence-specific contacts through the major groove. The β-IH motif (orange) is responsible for defining the single-stranded to double-stranded DNA boundary. The template strand and non-template strand of promoter DNA are represented as purple and pink ribbons, respectively.

A comparison of the sequences and structures required for mini-vRNAP and T7 RNAP promoter recognition, shown in Figure 4, is striking. T7 RNAP recognizes a bipartite double-stranded DNA sequence that spans from −17 to +6. Bases −17 to −5 constitute the upstream binding sequence, while bases −4 to +6 constitute the initiation region [41,42,43,44]. vRNAP, in contrast, recognizes a conserved promoter-hairpin structure along with sequences within it and its 3 bp loop (Figure 4a) [21,22,27].

Despite these drastic differences in promoter architecture, the RNAP motifs responsible for their recognition are remarkably similar (Figure 4b). Both RNAPs have a specificity loop that makes sequence-specific contacts with the promoter through the major groove, a β-IH structure responsible for defining the double-stranded to single-stranded DNA junction, and an upstream recognition element (−11 recognition element in mini-vRNAP and AT-rich recognition loop in T7 RNAP) [36,38,39,40]. Although the upstream recognition elements are more complex in mini-vRNAP, they share common structures nonetheless. This suggests that T7-like RNAPs developed unique strategies using the same set of basic tools to recognize their cognate promoters and ensure specificity.

**Figure 5:**
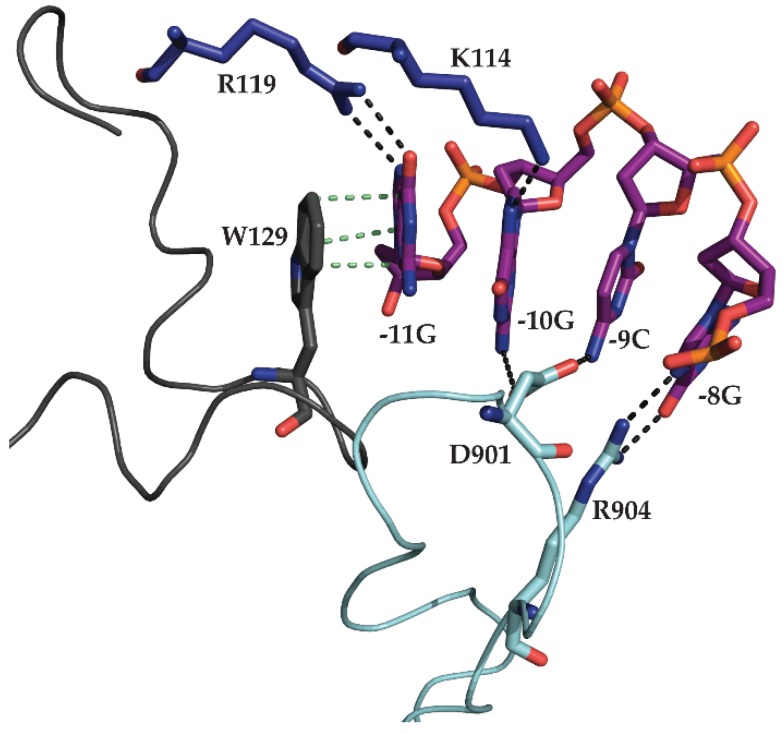
Summary of all sequence specific contacts required for mini-vRNAP recognition of early promoter P2. Promoter DNA represented as purple sticks. Mini-vRNAP structural motifs involved in recognition are represented as ribbons, with individual residues highlighted as sticks. Direct interactions are represented as dark grey (hydrogen bonds) or green (base stacking) dashed lines. W129 of the −11 recognition element (light grey) base-stacks with the −11G residue in the hairpin loop. Fingers domain (dark blue) residues K114 and R119 hydrogen bond with −10G and −11G, respectively. Specificity loop (cyan) residues D901 and R904 contact −9C/−10G and −8G through the major groove of the hairpin stem, respectively.

The N-terminal −11 recognition element contacts bases in the conserved loop through hydrogen bonding between K114 and R119 with −10G and −11G, respectively. The structure confirmed W129 base stacking with −11G [28,36,40]. The conformation of the hairpin loop explains its remarkable stability. Bases −9, −10, and −11 stack together and a sharp turn in DNA between bases −11 and −12 enables further base stacking interactions between −12 and −13 bases, while −10G and −12A form a sheared 5'G:A3' base pair. This unusual DNA structure explains both the sequence conservation and the remarkable stability of the promoter hairpin [22,25,26,27,40]. Three other elements are involved in promoter recognition. The β-IH defines the junction between double-stranded and single-stranded DNA between bases −5 and −4. In addition to defining the junction, this element enforces it by melting the 2 bps at the base of the 7 bp stem (structure P2_7a) with residues K267 and K268. Furthermore, specificity loop residues D901 and R904 make contacts through the major groove of the hairpin stem with −9C/−10G and −8G, respectively. Finally, finger residues K849 and K850 form salt bridges with the phosphate backbone between residues −12 and −13. Interestingly, there are no base contacts with the four As near the initiation site, suggesting that these bases act as a molecular ruler to start transcription eight nucleotides downstream of the −8 position [40]. These contacts, summarized in Figure 5, aid in the recognition of the promoter hairpin and render mini-vRNAP competent for transcription initiation.

**Figure 6:**
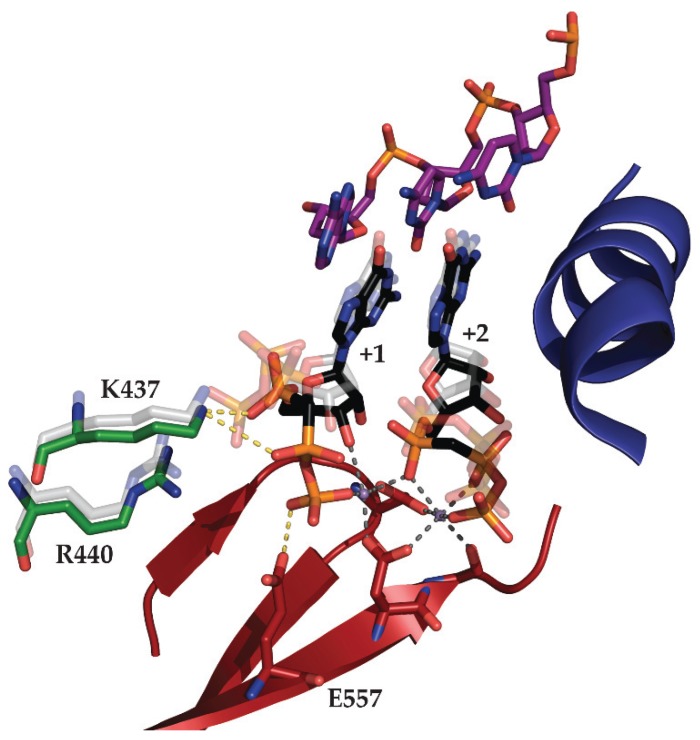
Structural transitions between mini-vRNAP SCI (Accession number 3Q22) and SCII (Accession number 3Q23) complexes required for stabilization of the initiating nucleotide. SCII complex structural motifs required for catalysis, O helix (dark blue) and motifs A and C (dark red), are represented as cartoons. Template strand DNA from −1 to +2 is represented as purple sticks. +1 and +2 GMPCPP molecules are represented as black sticks. Catalytic metals are represented as violet spheres. Palm domain residues required for stabilization of +1 NTP phosphate interactions are represented as green sticks. Catalytic aspartates and motif A residue E557 required for stabilization of +1 NTP phosphates are represented as red sticks. Interactions between residues and the catalytic aspartates are represented as dark grey dashes and interactions required for stabilization of +1 NTP phosphates are represented as yellow dashes. SCI residues K437 and R440, along with +1 and +2 GTP, are represented as transparent light grey sticks. Upon transition to the SCII complex, the phosphate residues of the initiating nucleotide break contact with R440 and are re-stabilized by E557 after a large conformational change. This change facilitates the repositioning of the 3'OH within 3.1 Å of the +2 NTP αP.

A unique aspect of DNA-dependent RNAPs is the ability to carry out first dinucleotide bond formation. A previous structural study using T7 RNAP failed to capture this event since the catalytic metal ion was absent and the substrate reactive groups were misaligned due to the use of the substrate analog 3'-deoxyGTP [45]. In an attempt to visualize this event with substrates containing all pertinent functional groups, GTP or the nonhydrolysable analog guanosine-5'-[(α,β)-methyleno] triphosphate (GMPCPP) and Mg^2+^ were soaked into preformed mini-vRNAP binary complex crystals [46]. Four structures were solved: (i) the precatalytic complex I (SCI) with two GTP molecules aligned with DNA bases +1 and +2 along with Mg^2+^ as the nucleotide-binding metal; (ii) the precatalytic complex II (SCII) with two GMPCPP molecules aligned with DNA bases +1 and +2 along with two Mn^2+^ catalytic metals; (iii) a mismatch complex (MC) with a GTP aligned with DNA base +1, but misaligned with the DNA base T at the +2 site along with two Mg^2+^ catalytic metals, and (iv) the product complex (PC) with a 2-mer RNA product and PPi [46]. These structures show that the initiating nucleotide base-stacks with the purine at the −1 position of the template strand, explaining the conservation of purines at the −1 position of many T7-like RNAP promoters. Secondly, the structure revealed for the first time the interactions of the initiating nucleotide with RNAPs: the palm residues K437 and R440 stabilize the binding of the initiating nucleotide [46]. Both catalytic metal ions, brought in along with the nucleotide substrates, cause a conformational shift in the catalytic aspartates and reorganization of the phosphates in the +1 NTP to break contact with R440 and initiate new contacts with E557, positioning the 3'OH 3.1 Å from the +2 NTP αP (Figure 6) [46]. In the product structure, the catalytic Mg^2+^s leave the active site and the catalytic aspartates shift back to their binary complex states [46]. These structures define the contacts required for coordination of the initiating nucleotide phosphate groups in the process and suggest that binding of the catalytic metal is the last step before catalysis of the dinucleotide bond. This notion has been supported by direct observation through time-resolved X-ray crystallographic studies [47].

### 2.2. N4 RNAPII Synthesizes N4 Middle RNAs

The existence of a second N4-encoded transcriptional activity was postulated based on the 100-fold decrease in the rate of post-infection RNA synthesis when cells were pretreated with chloramphenicol [10,11,48]. Therefore, most phage transcription requires the synthesis of N4 early gene products. Indeed, infection with N4 phage containing mutations in ORF15 (N4*am*15) or ORF16 (N4*am*23) mimics the chloramphenicol-pretreated wild type phage-infected cell phenotype, indicating that the products of these genes (gp15 and gp16) encode a second transcriptional activity responsible for middle transcription [10,11,48,49].

Attempts to purify the phage proteins responsible for this activity proved difficult due to their tight association with the host cytoplasmic membrane [48,50]. Characterization was facilitated by the discovery, through additional mutagenesis screens, of a third N4 gene product (gp2) required for middle transcription. N4*am*126S mutant phage, which has a mutation in ORF2, was defective in middle transcription but could complement infections by either N4ORF15*am* or N4ORF16*am* mutants, indicating the involvement of gp2 in this process [51]. An *in vitro* complementation assay was developed where gp15 and gp16 present in the supernatant of N4*am*126S-infected cells were combined with gp2 and template DNA present in the membrane fraction of N4ORF15*am-* or N4ORF16*am-*infected cells [51,52]. This assay was competent for *in vitro* RNA synthesis and allowed for the purification and characterization of N4 RNAPII (gp15–gp16).

Purified N4 RNAPII, a heterodimer of gp15 and gp16, could utilize denatured DNA or the gp2-DNA bound membrane complex as templates [52]. Transcription originating from denatured DNA displayed no sequence specificity, while *in vivo* and *in vitro* transcription from the gp2-membrane complex displayed increased specificity and activity. This suggests that gp2 may have a role in providing specificity to N4 RNAPII either by direct interaction or providing a specific secondary structure for promoter recognition in a sequence-dependent fashion [52,53]. Further *in vitro* studies showed that N4 RNAPII binds only to single-stranded DNA with no sequence specificity while addition of a high salt-wash from N4-infected cell membranes containing gp2 provided some specificity in runoff transcription assays [53]. Sites of N4 RNAPII transcription initiation were mapped to a series of overlapping transcripts confined to the leftmost 50% of the genome [19,54]. N4 RNAPII recognizes a minimal promoter spanning −7 to +2 that is highly enriched in AT pairs, which may be an important factor in promoter melting [54,55].

Sequence analysis of ORF15 and ORF16 (gp15 and gp16) showed that these proteins align to non-overlapping portions of T7 RNAP (883 aa), confirming that N4 RNAPII is a heterodimer and identifies the enzyme as a member of the T7-like “single-subunit” RNAP family [35,49,52]. Together, gp15 and gp16 contain all four conserved motifs and thirteen blocks of conserved sequence within members of the T7-like RNAP family across both subunits. Gp15 (269 aa) aligns to the N-terminus, thumb, and DX_2_GR domains, while gp16 (404 aa) aligns to the palm and fingers domains along with motifs A, B, and C [49,56]. N4 RNAPII (673 aa) represents a rather minimal RNAP, as it is one of the smallest members of this family, with a truncated thumb domain and a truncated N-terminal domain shown to be involved in promoter recognition in vRNAP and T7 RNAP [49]. However, N4 RNAPII shows much greater sequence homology to T7 RNAP than vRNAP and likely shares a similar architecture [34,49].

**Figure 7:**
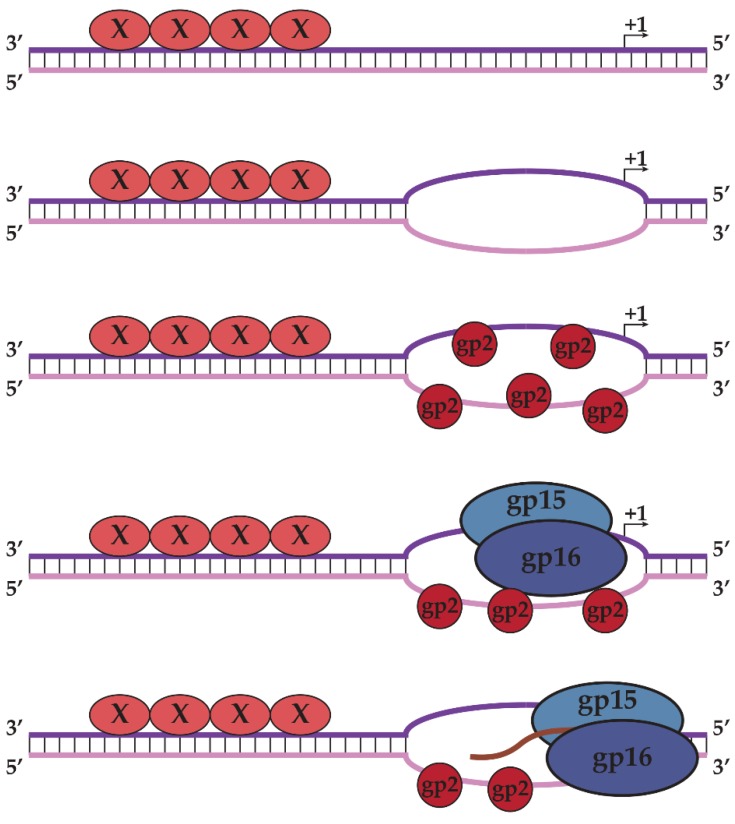
Model of middle transcription by N4 RNAPII. AT-rich double-stranded promoters are specifically recognized and melted by an unknown protein “X,” allowing gp2 to bind to single-stranded DNA. Gp2 recruits N4 RNAPII to the promoter through direct interactions, allowing N4 RNAPII to recognize specific sequences in the template strand and initiate transcription. Template and non-template strands of promoter DNA are represented as purple and pink, respectively.

To elucidate the role of gp2 in middle transcription, ORF2 was cloned and sequenced, revealing no homology to proteins of known function [57]. The purified protein was tested for DNA binding and transcription-enhancing properties *in vitro*. Surprisingly, gp2 was shown to be a single-stranded DNA-binding protein exhibiting no sequence specificity [57]. It activates transcription through a recruitment mechanism, as binding of gp2 to single-stranded DNA increased the affinity of N4 RNAPII for the same template. Recruitment occurs through binding to N4 RNAPII; the N4 RNAPII-gp2 complex withstands treatment with high salt [57]. The data presented above has led us to propose a model for middle transcription whereby an unknown protein binds to double-stranded DNA to unwind the AT-rich promoter sequence, allowing gp2 to bind to the single-stranded DNA at middle promoters and recruit N4 RNAPII through direct interactions to the promoter sequences. N4 RNAPII then recognizes specific sequences in the template strand and initiates transcription (Figure 7). Preliminary evidence suggests that the product of ORF1, gp1, is required for middle promoter utilization *in vivo*. Experiments to characterize gp1’s role in middle transcription and define the contacts required for N4 RNAPII promoter recognition are currently in progress.

### 2.3. N4SSB Directs E. coli σ^70^-RNAP to Synthesize N4 Late RNAs

Although N4 encodes two rifampicin-resistant transcribing activities, neither progeny nor virion structural proteins are produced in the presence of rifampicin, which suggests that the rifampicin-sensitive host RNAP is required for late transcription [10,11,19]. Progeny production in a rifampicin-resistant host was unaffected by presence of the drug, indicating that the host RNAP is responsible for N4 late transcription. Late transcripts localize to the right half of the phage genome and extend with opposite (leftward) polarity of early and middle transcripts [19]. A product of middle transcription, the N4 single-stranded DNA-binding protein (N4SSB), plays a dual role in N4 development. N4SSB is essential for DNA replication [58,59,60]. Genetic and biochemical analyses indicate that the N4SSB single-stranded DNA binding activity is dispensable while residues at the C-terminus are essential for late transcription activation. N4SSB interacts with a highly conserved region at the C-terminus of the β' subunit of *E. coli* σ^70^-RNAP [61,62,63]. These findings reveal that bacteriophage N4 uses single-stranded DNA binding proteins *Eco*SSB, N4 gp2, N4SSB, as transcription activators. We surmise that the dependence on *Eco*SSB and gp2 derives from the interaction of vRNAP and N4 RNAPII with non-canonical DNA structures at the promoters, while N4SSB activation of late transcription couples phage morphogenesis to DNA replication.

## 3. N4 RNAPs Have Multiple Roles in Phage Development

Although we have focused on the roles of vRNAP and N4 RNAPII in transcription, their utility to N4 stretches across many developmental processes. The coding sequence of the vRNAP polypeptide (ORF50, 10.5 kbp) encompasses approximately 15% of the 72 kbp phage genome; its size and domain architecture suggest multiple functions during N4 development. vRNAP, present in four copies per virion, was localized above the portal protein by cryo-electron microscopy [64]. Based on this localization, injection of N4 genomic DNA must follow vRNAP out of capsids and into the host. Upon interaction of the N4 tail sheath protein (N4gp65) with *E. coli* outer membrane protein NfrA, a conformational change occurs in the phage tail that leads to the injection of the 3500 aa vRNAP through a 25 Å diameter tail tube and localization to the host cytoplasmic membrane [48,50,64,65,66,67,68]. Movement of vRNAP away from the portal allows the first 500 bp of the N4 genome to enter the host, where it is acted on by host DNA gyrase [14,64,69]. This enzyme introduces negative supercoils into N4 genomic DNA, leading to the extrusion of promoter hairpins [25]. vRNAP recognizes the promoter structure, which induces a structural change to activate the RNAP for transcription initiation [36,40]. Transcription of early genes leads to the injection of the genome, which is completed only upon the synthesis of the middle transcriptional machinery responsible for the synthesis of proteins required for N4 DNA replication [69]. One such product, N4SSB, interacts with the host σ^70^-RNAP redirecting it to late promoters [62].

The developmental scheme outlined implicates a role of N4 transcriptional machineries in encapsidation, genome injection, transcription, and DNA replication. The encapsidation of its own early RNAP has distinct advantages for this phage in overcoming barriers of infection. N4, a member of the *Podoviridae* family, has a short, non-contractile tail incapable of traversing the host periplasm and injecting the DNA into the cytoplasm. vRNAP may have a role in overcoming this barrier, as suggested by its membrane association and role in genome injection [50,69]. Furthermore, the injection of genomic DNA in an inactive conformation provides a checkpoint for N4 phage to sample the energy state of the host by reading out the [ATP]/[ADP] ratio through the ATP-dependent activity of host DNA gyrase required for hairpin extrusion and early promoter activation [17,70,71]. The injection of vRNAP in an inactive conformation also precludes its interaction with host DNA, which is in greater abundance than the single copy of phage DNA. These mechanisms are but some of the many strategies employed by bacteriophage N4 to maximize progeny yield [72,73,74].

To our knowledge, N4 is the only bacteriophage that utilizes three different RNAPs to regulate the expression of its genes. Why does N4 use this strategy? The requirement for supercoil-dependent formation of the hairpin structure at the N4 vRNAP promoters concurrent with genome injection constrains their localization to the left end of the genome, which is injected into the host first. Moreover, the four vRNAP molecules injected from virions are insufficient for transcription from the large number of promoters. Therefore, to utilize all middle promoters and increase middle transcript abundance, additional polymerases must be synthesized. Synthesizing the 320 kDa vRNAP, with two large domains inessential for transcription, would waste resources where the minimal 80 kDa N4 RNAPII protein suffices. Why encode RNAPs required for early and middle transcription while relying on the host RNAP for late transcription in a pattern opposite of any other well-studied phage? Hijacking of host polymerase severely reduces host transcription, diverting resources to N4. Utilizing the N4SSB protein required for N4 DNA replication ensures that the transcription of late genes involved in virion assembly and lysis does not proceed until genome replication has begun. The ability to simultaneously transcribe the genes required for genome replication and morphogenetic proteins using two distinct transcriptional machineries allows for the production of 3000 progeny per infected bacterium [75].

## 4. Phylogenetic Analysis of N4-Like Phage Proteins Involved in Transcription

Original studies in 2002 suggested that vRNAP and N4 RNAPII are highly divergent members of the T7-like RNAP family with very little sequence homology to any other known phage RNAPs [34,49]. With the advent of next-generation sequencing technologies, there has been a large influx of phage genomes annotated and submitted in the past 10 years [76]. As a consequence, we are now able to identify a plethora of new phage and expand our knowledge of T7-like RNAP diversity.

**Figure 8:**
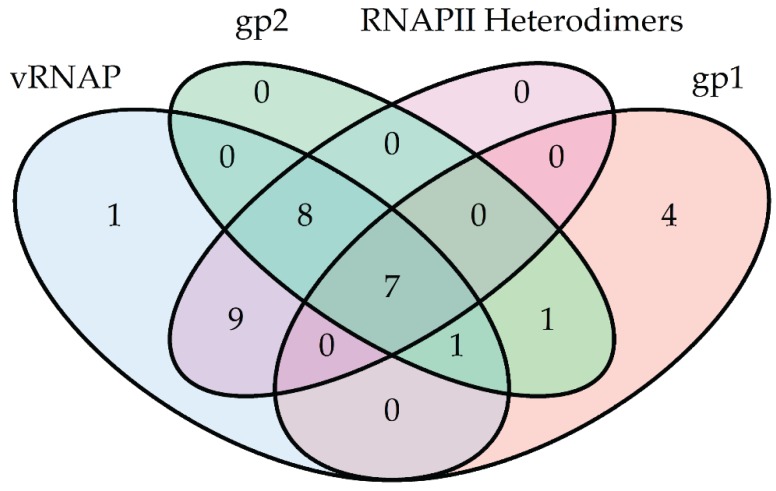
Venn diagram of species containing homologs of N4-encoded early and middle transcriptional machinery proteins vRNAP (blue), gp2 (green), gp1 (pink), and N4 RNAPII (purple) identified by DELTA-BLAST of NCBI RefSeq proteins. For simplicity, the category of species containing single-subunit RNAPs homologous to N4 RNAPII has been excluded. The number of species within each cross-section is indicated.

Using this expanded dataset, homologs of vRNAP, N4 RNAPII, gp1, and gp2 were identified by DELTA-BLAST and subjected to maximum-likelihood neighbor-joining phylogenetic analyses using the software program MEGA6 [77]. We detected over 500 homologs of N4 RNAPII and 26 homologs of vRNAP. Of the >500 N4 RNAPII homologs, only 24 are heterodimers, while the remainders are single-subunit RNAPs. Sequence alignments show significantly greater homology to gp16 than gp15, which is not surprising given the localization of motifs A, B, and C in gp16 while gp15 is truncated and contains the more variable promoter recognition elements. Interestingly, all species possessing a virion-encapsidated RNAP homolog, except two, also possess a heterodimeric RNAP. The exceptions have sequences similar to gp16 split into two separate genes, highlighting the difficulty of using unannotated genomes. By parsimony, we infer that virion-encapsidated RNAPs predate heterodimeric T7-like RNAPs. Furthermore, 13 and 17 species contain annotated homologs of the middle transcription cofactors gp1 and gp2, respectively. Of these species, seven show absolute conservation of the early and middle transcriptional machineries (Figure 8). This suggests a phylogenetic relationship between virion-encapsidated and heterodimeric RNAP transcriptional paradigms.

The origin and advantage of having a heterodimeric versus single-subunit RNAP remain unclear. Sequence alignment analysis of all heterodimeric RNAPs was performed and compared to the T7 RNAP sequence in an attempt to identify the dimerization interface. Several blocks of sequence homology in the C-terminus of gp15 and N-terminus of gp16 that do not overlap with motifs required for catalysis were identified. These blocks map to a three-helix bundle at the base of the T7 RNAP palm and may represent a dimerization domain required for heterodimer stability.

Finally, sequence analysis of the N- and C-terminal domains of vRNAP-like proteins revealed several blocks of sequence conservation. These sequence blocks may help to define the regions of each domain required for encapsidation and genome injection [78].

## 5. Conclusions

T7 RNAP, considered the founding member and prototype of the single-subunit family of RNAPs, catalyzes faithful initiation, elongation and termination of transcription independent of other phage or host proteins. However, biochemical and genetic characterization of other members of the family (vRNAP, N4 RNAPII, human and yeast mitochondrial RNAPs) show that these RNAPs rely on transcription factors for accurate initiation and/or elongation [35,79,80,81]. Therefore, T7 RNAP’s property of transcriptional independence is the exception, rather than the rule (Table 1).

**Table 1:** T7-like RNAPs and their required cofactors.

Organism	RNAP	Cofactor(s)
T7	T7 RNAP	None
*Saccharomyces cerevisiae* mitochondria	Rpo41	Mtf1p
*Homo sapiens* mitochondria	POLRMT	TFAM and TFB2M
N4	N4 vRNAP	*Eco*SSB
N4	N4 RNAPII	gp1 and gp2

Through studying N4 RNAPs, we have expanded the diversity and knowledge of the T7-like “single-subunit” RNAPs, come to a new understanding of the importance of regulation of transcription through changes in DNA conformation, developed a model for understanding the role of cofactors in transcription initiation, expanded on the molecular basis of promoter recognition and transcription initiation, and discovered multiple mechanisms by which transcriptional machinery can be used to overcome barriers of infection.

Although the N4 transcriptional machinery provides a framework for understanding the importance of transcription in phage development, several open questions remain. Current work is focused on understanding the role that N4 RNAPII cofactors have on DNA conformation, how cofactors interact with the RNAP to initiate transcription, and whether these interactions are conserved among RNAPs such as POLRMT and Rpo41, which also rely on cofactors for transcription. We are also interested in exploring how conserved RNAP motifs involved in promoter recognition have evolved to recognize such a wide variety of promoter sequences and structures. Finally, the presence of the vRNAP polypeptide in virions and its involvement in genome injection provides a unique manipulable system to investigate the process of protein and genome injection into the host.
